# Human Papillomavirus Infection in 674 Chinese Patients with Laryngeal Squamous Cell Carcinoma

**DOI:** 10.1371/journal.pone.0115914

**Published:** 2014-12-23

**Authors:** Yanan Xu, Suru Liu, Hongliang Yi, Jiadong Wang, Pin Dong, Xiaoyan Li, Shankai Yin

**Affiliations:** 1 Department of Otolaryngology, Shanghai Jiao Tong University Affiliated Sixth People's Hospital, Shanghai, China; 2 Department of Head and Neck Surgery, Renji Hospital of Shanghai Jiao Tong University School of Medicine, Shanghai, China; 3 Department of Otolaryngology Head and Neck Surgery, Shanghai Jiao Tong University Affiliated First People's Hospital, Shanghai, China; 4 Otolaryngological Institute of Shanghai Jiaotong University, Shanghai, China; 5 Department of Otolaryngology Head and Neck Surgery, Children's Hospital Affiliated to Shanghai Jiaotong University, Shanghai, China; Georgetown University, United States of America

## Abstract

**Objectives:**

Previous reports suggest a strong association between human papillomavirus (HPV) and the etiology of laryngeal squamous cell carcinoma (LSCC). However, clinical data regarding the HPV infection rate among LSCC patients remain largely inconsistent.

**Methods:**

In total, 674 LSCC patients from three major hospitals in Shanghai were enrolled in this study. We determined the patients' HPV infection status using immunohistochemistry and the GenoArray HPV genotyping assay and calculated their long-term survival rate using the Kaplan-Meier method.

**Results:**

The total P16-positive rate according to immunostaining results was 7.57% (51/674). None of the P16-negative patients were HPV-positive according to the HPV genotyping test. The rate of HPV infection among patients with LSCC was 4.9% (33/674). HPV infection was more common among nonsmokers (P<0.05), nondrinkers (P<0.05), and patients with supraglottic LSCC (P<0.05). Of the 33 HPV-positive patients, 28 (84.8%) were infected with HPV-16, 2 with HPV-18, 1 with HPV-31, 1 with HPV-33 and 1 with HPV-45. The 3-year overall survival rate and progression-free survival rate were higher in HPV-positive than HPV-negative patients, but the difference was not statistically significant (76.3% vs. 70.7%, P = 0.30 and 65.1% vs. 58.3%, P = 0.37, respectively).

**Conclusion:**

HPV was not a main causal factor in LSCC carcinogenesis in this Chinese population. HPV infection did not alter patients' overall survival or progression-free survival rates in this study.

## Introduction

Cancer of the larynx accounts for >3% of all cancers, making it the sixth most common cancer worldwide [1]. In 2012, an estimated 3650 laryngeal cancer-related deaths occurred globally [1].

The established risk factors for laryngeal squamous cell carcinoma (LSCC) are tobacco and alcohol abuse; however, molecular evidence has supported a role for human papillomavirus (HPV), particularly HPV-16, in the pathogenesis of LSCC [2]. Previous reports also show a strong association between HPV infection and LSCC carcinogenesis. However, clinical data on the HPV infection rate among patients with LSCC remain largely inconsistent, ranging from 0% to 85% [3], [4]. This disparity is due primarily to geographical differences among the studies conducted and inadequate separation of patients with laryngeal carcinoma from patients with other cancers of the head-and-neck region, such as oropharyngeal squamous cell carcinoma (OPSCC) [5]. Additionally, differences in the analytical sensitivity and specificity of HPV genotyping methods, the limited spectrum of HPV types analyzed, and differences among HPV diagnostic criteria [6], [7] have contributed to the inconsistent results regarding HPV infection in patients with LSCC.

Identification of a high viral infection rate for a given cancer may facilitate the use of preventive viral vaccination. Genomic DNA of oncogenic HPV is detected in 72% of patients with OPSCC [7]. The International Agency for Research on Cancer reported that HPV-16 causes cancer of the oropharynx [8]. With the introduction of the HPV quadrivalent vaccine (Gardasil; Merck) and bivalent vaccine (Cervarix; GlaxoSmithKline) for the prevention of cervical carcinoma [9], the same preventive approach in the form of vaccination of adolescents of both sexes has been suggested for non-anogenital cancers, such as head-and-neck squamous cell carcinoma (HNSCC) [10]. This is particularly important because the worldwide incidence and mortality rates of HNSCC are higher than those of cervical cancer [1].

Viral infection is also an important confounding factor for cancer prognosis. Several reports have suggested HPV-positive HNSCC, notably HNSCC that arises from oropharyngeal sites and integrates HPV-16, has a better prognosis than does HPV-negative HNSCC [11]–[13]. This clinical entity may be attributable to enhanced sensitivity to treatment due to a wild-type TP53, allowing for an apoptotic response of cancer cells to radiation and chemoradiation [14]. Few survival analyses have isolated LSCC from other cancers of the head and neck region. Thus, the prognosis of HPV-positive LSCC has yet to be determined.

We therefore conducted the current study, which focused exclusively on LSCC. Our aim was to evaluate the HPV infection rate in Chinese patients with LSCC and assess the prognostic value of HPV-associated LSCC.

## Methods

### Patients

This was a longitudinal cohort study (started April 2006) of patients with newly diagnosed LSCC in three hospitals: First People's Hospital, Sixth People's Hospital, and Renji Hospital. Patients with a confirmed pathological diagnosis of LSCC were recruited. No patients in our series that had a previous history of HPV related OPSCC and cervical cancer.

The study protocol was approved by the Research Ethics Committees of First People's Hospital, Sixth People's Hospital, and Renji Hospital. Written informed consent was obtained from all patients.

### Data collection

The demographic data and clinical and pathological characteristics of 674 patients with LSCC were obtained from an electronic medical record system used in each hospital. The TNM staging system was used to classify LSCC in accordance with the American Joint Committee on Cancer classification. Detailed information is provided in Table 1. No patients had distant metastasis.

**Table 1:** Explanatory variables for 674 patients with LSCC in three hospitals.

	Renji Hospital	Sixth People's Hospital	First People's Hospital	P
	N = 268	N = 159	N = 247	
	N (%)			
Sex				
Female	5 (1.9)	4 (2.5)	11 (4.5)	0.21
Male	263 (98.1)	155 (97.5)	236 (95.6)	
Age in years				
Median [range]	60.9 [37–84]	64.3 [39–84]	62.3 [21–82]	0.54
Tobacco				
Smokers	221 (82.5)	137 (86.2)	201 (81.4)	0.44
Nonsmokers	47 (17.5)	22 (13.8)	46 (18.6)	
Alcohol				
Drinkers	191 (68.7)	92 (61.7)	155 (62.8)	0.23
Nondrinkers	77 (31.3)	67 (38.3)	92 (37.3)	
Tumor location				
Supraglottic	60 (22.4)	39 (24.5)	73 (29.6)	0.21
Glottic	194 (72.4)	116 (73.0)	161 (65.2)	
Subglottic	14 (5.2)	4 (2.5)	13 (5.3)	
Clinical tumor classification				
cT1	70 (26.1)	39 (24.5)	53 (21.5)	0.43
cT2	101 (37.7)	54 (34.0)	109 (44.1)	
cT3	55 (20.5)	41 (25.8)	49 (19.8)	
cT4	42 (15.7)	25 (15.8)	36 (14.6)	
Clinical lymph node classification				
cN0	157 (58.6)	78 (49.1)	128 (51.8)	0.29
cN1	20 (7.5)	22 (13.8)	23 (9.3)	
cN2	86 (32.1)	55 (34.6)	89 (36.0)	
cN3	5 (1.9)	4 (2.5)	7 (2.8)	

Patients' smoking and drinking histories were also available in our electronic medical record system. Patients were classified as smokers or nonsmokers and drinkers or nondrinkers. Smokers were defined as individuals who smoked at least once a week for 1 year [15]. Drinkers were defined as individuals who drank at least one 12-oz beer, one 6-oz glass of wine, one 3-oz mixed drink, or one 1.5-oz shot of liquor once per week for 1 year [15].

One representative formalin-fixed, paraffin-embedded (FFPE) block was retrieved for each case. Histological diagnosis of LSCC was confirmed by a pathologist.

### Treatment

**Figure 1:**
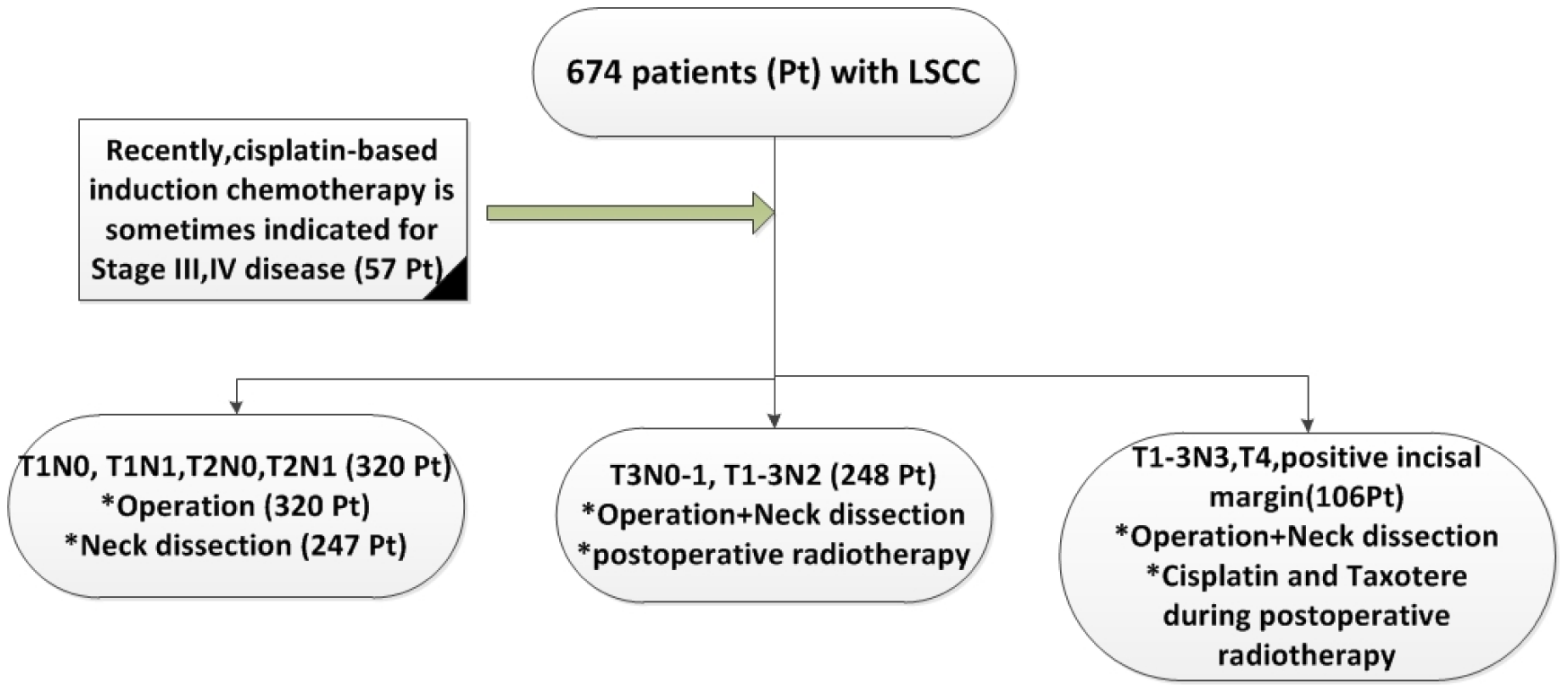
Treatment with curative intent received by 674 patients with LSCC.

Patients were treated according to the National Comprehensive Cancer Network Guidelines. The treatments are illustrated in Fig. 1. No treatments were based on the patients' HPV status because these markers are not routinely examined before treatment in these three centers.

### Follow-up

Patients were followed up every 3 months for the first 3 years, every 6 months for the fourth and fifth years, and every 12 months for the following years. The criteria for determining a disease-free state and identifying disease progression were as follows. Fiberoptic laryngoscopy or electric laryngoscopy and imaging examinations (CT, MRI, PET, chest X-ray, liver ultrasonography, and others) were performed to evaluate suspicious lesions. When suspicious lesions were observed, biopsies with subsequent pathological examination were obtained to determine if laryngeal recurrence and/or regional lymphatic or distant metastasis had occurred. A progression-free state was defined as the absence of cancer as demonstrated by laryngoscopy and imaging examinations and (if necessary) pathological examination following biopsy. Postoperative complications were not included in the definition of disease, which referred only to the presence, recurrence, or metastasis of cancer.

### P16 immunohistochemistry

**Figure 2:**
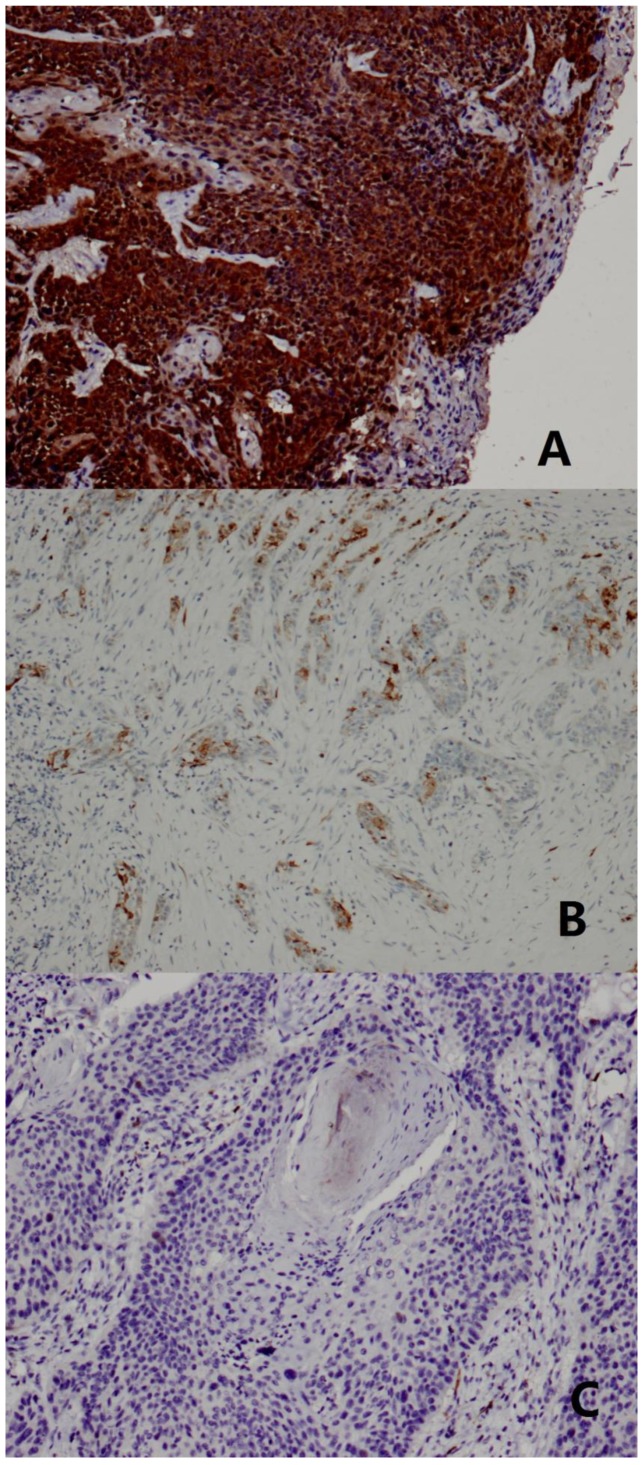
P16 immunohistochemistry. (**A**) Presence of HPV as visualized by strong cytoplasmic and nuclear staining for P16 by immunohistochemistry in >70% of the malignant cells. (**B**) Low P16 staining in <70% of the malignant cells was scored as negative. (**C**) Negative P16 staining. (×100).

Immunostaining for P16 was performed on representative 4-µm sections cut from FFPE tissue blocks that were obtained before patients received chemotherapy or radiotherapy. P16 immunohistochemistry was carried out using a proprietary kit (CINtec p16 Histology; Roche, mtm Laboratories) on a Roche Autostainer (Benchmark XT; Roche, Switzerland). An LSCC with high P16 expression was used as a positive control. The primary antibody was omitted from negative controls. P16 immunohistochemistry was scored as positive if there was strong and diffuse nuclear and cytoplasmic staining present in >70% of the malignant cells [16]. All other staining patterns were scored as negative (Fig. 2). All samples were scored independently by two senior head and neck pathologists. If there was any disagreement, a third senior pathologist would take part in discussion until consensus was reached.

### DNA extraction and assessment of sample adequacy

All tissue preparation was conducted in a Class II biological safety cabinet with new sterilized disposable consumables for each specimen to avoid cross-contamination. Sectioning was performed with appropriate precautions to prevent interblock DNA contamination. DNA was extracted from 2- ×10-µm sections of FFPE biopsies using a Qiagen FFPE kit (Hilden, Germany) according to the manufacturer's instructions. We used NanoDrop to measure the concentration and purity of DNA. Resulting DNA preparations were stored at −20°C.

### HPV GenoArray and genotyping assay

The GenoArray test by HybriBio (Chaozhou Hybribio Limited Corporation) is an L1 consensus primer-based PCR assay that is capable of amplifying 21 HPV genotypes, including 13 HR types (types 16, 18, 31, 33, 35, 39, 45, 51, 52, 56, 58, 59, and 68), 2 probable HR types (types 53 and 66), and 6 LR and unknown-risk types (types 6, 11, 42, 43, 44, and CP8304 [HPV-81]). The assay was performed according to the manufacturer's protocol [17].

### Statistical analysis

Multivariate logistic-regression models were used to estimate odds ratios and their associated 95% confidence intervals. Categorical variables were compared using a chi-squared test and Fisher's exact test. Differences were considered significant at P<0.05 in two-tailed tests. Overall survival analyses were based on the duration of time from the end of treatment to death; survivors were censored at their last follow-up. The Kaplan–Meier method was used for survival analysis. Data were analyzed using the SAS 9.3 software.

## Results

Tumors were classified as HPV-positive if they overexpressed P16 (as shown by immunohistochemisty) and contained HPV DNA (as shown by HPV genotyping) [18]. The total P16-positive rate according to immunostaining results was 7.57% (51/674), 6.29% (10/159) in Sixth People's Hospital, 7.46% (20/268) in Renji Hospital and 8.50% (21/247) in First People's Hospital (P>0.05). HPV infection was detected only in 33 patients (4.90%, 33/674) by HPV genotyping (Table 2). None of the P16-negative patients were HPV-positive according to the HPV genotyping test. HPV infection was more common in nonsmokers (10% nonsmokers vs. 4% smokers, P = 0.01), nondrinkers (7% nondrinkers vs. 4% drinkers, P = 0.04), and patients with supraglottic LSCC (9% supraglottic and 4% glottic vs. 0% subglottic, P = 0.03) (Table 2).

**Table 2:** Clinical parameters of 674 patients with LSCC according to HPV status.

Characteristic	Patients, n (%)		P
	HPV-positive	HPV-negative	
Sex			
Male	33 (5)	621 (95)	0.62
Female	0 (0)	20 (100)	
Age in years, median [range]	60.3 [21–84]	64.3 [46–75]	0.34
Tobacco			
Nonsmokers	11 (10)	104 (90)	0.01
Smokers	22 (4)	537 (96)	
Alcohol			
Nondrinkers	17 (7)	219 (93)	0.04
Drinkers	16 (4)	422 (96)	
Tumor location			
Supraglottic	15 (9)	157 (91)	0.03
Glottic	18 (4)	453 (96)	
Subglottic	0 (0)	31 (100)	
Clinical tumor classification			
cT1	8 (5)	154 (95)	0.18
cT2	16 (6)	248 (94)	
cT3	8 (6)	137 (94)	
cT4	1 (1)	102 (99)	
Clinical lymph node classification			
cN0	20 (6)	343 (94)	0.92
cN1	3 (5)	62 (95)	
cN2	10 (4)	222 (96)	
cN3	0 (0)	14 (100)	
Hospital			
First People's Hospital	14 (6)	233 (94)	0.69
Sixth People's Hospital	6 (4)	153 (96)	
Renji Hospital	13 (5)	255 (95)	

**Figure 3:**
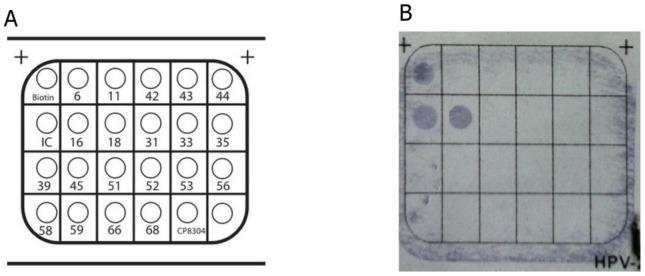
HPV GenoArray and genotyping assay. (**A**) The genotyping results were evaluated by means of a colorimetric change on the chip under direct visualization. Spots of “Biotin” indicate the hybridization-positive control gene, and “IC” indicates the inner control gene. (**B**) HPV-16 positive.

In total, 33 HPV-positive patients were identified using the GenoArray assay, which is capable of amplifying 21 HPV genotypes. Twenty-eight of these 33 patients (84.8%) were infected with HPV-16, 2 (6.1%) with HPV-18, 1 with HPV-31, 1 with HPV-33, and 1 with HPV-45 (Fig. 3).

**Figure 4:**
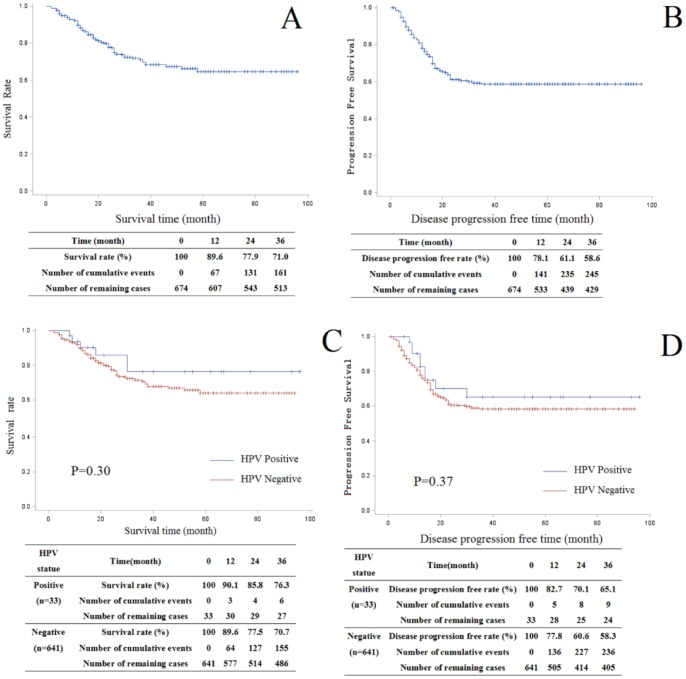
Kaplan–Meier overall survival and disease progression-free survival curves. (**A**) Overall survival rate for 674 patients with LSCC (**B**) Progression-free survival rate for 674 patients with LSCC. (**C**) Overall survival rate for HPV-positive (n = 33) and HPV-negative (n = 641) patients (P = 0.30). (**D**) Progression-free survival rate for HPV-positive (n = 33) and HPV-negative (n = 641) patients (P = 0.37).

For the survival analysis, the mean follow-up was 45.3 months (range, 2–96 months). The overall survival rates at 1, 2, and 3 years after treatment were 89.6%, 77.9%, and 71.0%, respectively. The progression-free survival rates at 1, 2, and 3 years after treatment were 78.1%, 61.1%, and 58.6%, respectively. The overall survival rates at 1, 2, and 3 years for HPV-positive patients were higher than those for HPV-negative patients (90.1%, 85.8%, and 76.3% vs. 89.6%, 77.5%, and 70.7% at 1, 2, and 3 years after treatment, respectively), but the differences were not statistically significant (P = 0.30). The progression-free rates for HPV-positive patients were also higher than those for HPV-negative patients (82.7%, 70.1%, and 65.1% vs. 77.8%, 60.6%, and 58.3% at 1, 2, and 3 years after treatment, respectively); however, the differences were not statistically significant (P = 0.37) (Fig. 4).

## Discussion

Our study showed all 33 HPV positive cases were P16 immunostaining positive (accounting for 100% P16 sensitivity). We also found 33 cases among 51 P16 positive patients were HPV positive (accounting for 65% P16 specificity). These results are similar to those obtained by Smeets et al. [19], who found a P16 immunostaining sensitivity of 100% and specificity of 79%. We confirmed that P16 immunostaining is a good first-step assay for elimination of HPV-negative cases from subsequent analyses.

The present study is the first to elucidate the HPV infection rate in patients with LSCC from 3 major hospitals in Shanghai, China. The 4.9% HPV infection rate suggests that HPV is not a main causal factor in LSCC carcinogenesis in this Chinese population, unlike for OPSCC. HPV-16 was a common HPV type (84.8%) in our study as well as in most previous studies on HNSCC [11], [20].

A recent meta-analysis [3] showed an overall HPV infection rate of 28.0% in patients with LSCC and a strong association between HPV infection and LSCC risk; the OR was 5.39 (95% CI, 3.32–8.73), which is even higher than that for OPSCC (OR, 4.3; 95% CI, 2.1–8.9) [21]. Our study showed a markedly lower HPV infection rate than those reported in the above meta-analysis. Multiple factors contribute to the discrepancy in the HPV infection rates. For example, patient selection (geographical differences of the patient, tobacco and alcohol status of the patient, tumor location of the patient); different measures used in individual studies and spectrum of HPV types analyzed.

The HPV prevalence determined in our study is consistent with the findings of several studies on LSCC from Germany [4] and France [22], in which the HPV infection rates were <5.0% and 3.8%, respectively. However, the HPV prevalence in the present study is lower than that found in two studies from the United States [5], [23] (23.7% and 24.0%, respectively). Li [24] found that 0 of 16 tonsil cancer specimens from Chinese patients were HPV-DNA–positive, whereas those from Australia had a positivity rate of 46% using the same test method. Bruni et al. [25] analyzed 194 studies of cervical HPV infection in women with normal cytological findings and reported that the HPV infection rates varied geographically, with African and Latin American regions showing higher average HPV infection rates than Eastern Asian regions (33.6%, 35.4%, and 10.7%, respectively). Therefore, ethnicity may have played a role in the low HPV infection rate in patients with LSCC in our study.

Traditionally, smoking and excessive alcohol consumption have been considered to be the main risk factors for LSCC. Tobacco and HPV infection can cause HNSCC through a similar mechanism, namely by inactivation of host oncosuppressor genes. It is therefore conceivable that in the United States, where smoking has been declining for decades, a substantial fraction of HNSCC attributable to HPV infection is now detected among nonsmokers or former smokers [5].

Two recent large-scale exome sequencing projects in patients with HNSCC [26], [27] revealed that HPV-negative tumors accumulate at least twice as many mutations. The carcinogenesis of HPV-negative HNSCC is based on acquisition of a large number of mutations in many different signaling pathways [26]. In contrast, the carcinogenesis of HPV-positive tumors is modulated by the activities of E6/E7 viral oncoproteins. An association between HPV-positive, P16-positive OPSCC and survival outcomes was reported in a retrospective analysis of 800 patients; the study concluded that patients with HPV-positive OPSCC have a better prognosis than do patients with HPV-negative cancers [28]. Further studies [5], [23] found that only patients with HPV-positive tumors in oropharyngeal sites showed significantly better survival than did HPV-negative patients; no survival benefit was observed for non-oropharyngeal tumors. In the current study, the overall survival and disease progression-free survival among all 674 patients with LSCC were better in HPV-positive patients than in HPV-negative patients, although the difference was not statistically significant. This is consistent with recent studies of HNSCC that reported that the HPV status is not a prognostic marker for hypopharyngeal SCC [29] or oropharyngeal SCC outside the tonsils and base of the tongue [30].

In conclusion, we found that the HPV infection rate in patients with LSCC was 4.9%, suggesting that HPV is not a main causal factor in LSCC carcinogenesis in this Chinese population. HPV infection did not alter patients' overall survival or disease progression-free survival rates in this study.
